# Copper Nanoparticle Loaded Electrospun Patches for Infected Wound Treatment: From Development to In-Vivo Application

**DOI:** 10.3390/polym16192733

**Published:** 2024-09-27

**Authors:** Anna Butsyk, Yulia Varava, Roman Moskalenko, Yevheniia Husak, Artem Piddubnyi, Anastasiia Denysenko, Valeriia Korniienko, Agne Ramanaviciute, Rafal Banasiuk, Maksym Pogorielov, Arunas Ramanavicius, Viktoriia Korniienko

**Affiliations:** 1 Ukrainian-Swedish Research Center SUMEYA, Medical Institute, Sumy State University, 116, Kharkivska Str., 40007 Sumy, Ukraine; a.butsyk@med.sumdu.edu.ua (A.B.); a.piddubny@med.sumdu.edu.ua (A.P.); 2 Biomedical Research Centre, Medical Institute, Sumy State University, 116, Kharkivska Str., 40007 Sumy, Ukraine; yuliia.varava@gmail.com (Y.V.); a.denysenko@med.sumdu.edu.ua (A.D.); kornienvaleria18@gmail.com (V.K.); maksym.pogorielov@lu.lv (M.P.); 3 Faculty of Chemistry, Silesian University of Technology, 44-100 Gliwice, Poland; yevheniia.husak@polsl.pl; 4 Department of Physical Chemistry, Institute of Chemistry, Faculty of Chemistry and Geosciences, Vilnius University, Naugarduko Str. 24, LT-03225 Vilnius, Lithuania; agne.ramanaviciute@gmail.com; 5 NanoWave, 02-676 Warsaw, Poland; banasiuk@gmail.com; 6 Institute of Atomic Physics and Spectroscopy, University of Latvia, 3 Jelgavas Str., LV-1004 Riga, Latvia

**Keywords:** infected wound, electrospinning, wound patches, polylactic acid, chitosan, copper nanoparticles, nanomedicine, *Staphylococcus aureus*, *Escherichia coli*, *Pseudomonas aeruginosa*

## Abstract

This study investigates the development and application of electrospun wound dressings based on polylactic acid (PLA) nanofibers, chitosan, and copper nanoparticles (CuNPs) for the treatment of purulent skin wounds. The materials were evaluated for their structural, antibacterial, and wound healing properties using an animal model. PLA/Ch-CuNPs demonstrated the most significant antibacterial activity against *Staphylococcus aureus*, *Escherichia coli*, and *Pseudomonas aeruginosa*, surpassing the other tested materials. The integration of CuNPs into the nanofiber matrices not only enhanced the antimicrobial efficacy but also maintained the structural integrity and biocompatibility of the dressings. In vivo experiments using a rat model showed that PLA/Ch-CuNPs facilitated faster wound healing with reduced exudative and inflammatory responses compared to PLA alone or PLA-CuNPs. Histological and immunohistochemical assessments revealed that the combination of PLA, chitosan, and CuNPs mitigated the inflammatory processes and promoted tissue regeneration more effectively. However, this study identified potential toxicity related to copper ions, emphasizing the need for careful optimization of CuNP concentrations. These findings suggest that PLA/Ch-CuNPs could serve as a potent, cost-effective wound dressing with broad-spectrum antibacterial properties, addressing the challenge of antibiotic-resistant infections and enhancing wound healing outcomes.

## 1. Introduction

Purulent skin wounds have emerged as a significant public health challenge worldwide. The increasing number of patients with these wounds places a considerable burden on healthcare systems, exacerbating issues like antibiotic resistance and chronic inflammation. Skin wounds are frequently infected by bacteria such as *Staphylococcus aureus* (*S. aureus*), with growing concern over the rapid spread of methicillin-resistant *S. aureus* (MRSA) [1]. The treatment of wounds associated with antibiotic-resistant microorganisms limits standard treatment approaches, prolonging healing time and often resulting in long-term chronic inflammation [2].

Effective skin wound treatment encompasses proper cleaning, infection control, and the protection of the wound surface with dressing materials, which are crucial in managing purulent skin infections. These dressings can be of natural or synthetic origins, with the latter being synthesized using various methods [3]. Electrospinning (ES) is a prominent technique for preparing ultra-thin nanofiber scaffolds. Dressings made via electrospinning exhibit high porosity, mechanical durability, and remarkable biocompatibility [4]. Moreover, these nanofibers can be loaded with bioactive materials, such as nanoparticles, to promote antimicrobial activity and thus reduce the wound healing time [5].

Metal and metal-based nanoparticles, particularly silver [6], gold [7], and zinc [8], are widely used in biomedical applications due to their unique antimicrobial action, high surface-to-volume ratio, enhanced reactivity and interaction within biological systems, and biocompatibility. Recently, copper nanoparticles (CuNPs) have garnered significant attention. Copper is an essential inorganic element in the human body, serving as a cofactor for several enzymes involved in iron oxidation, proper hemoglobin synthesis, collagen and elastin cross-linking, immune cell production and function, and angiogenesis [9,10,11]. CuNPs are promising candidates for various technological applications [12]. Some of the especially promising CuNPs application areas are related to biomedicine, due to their antimicrobial, antiviral, and antifungal activity, their cost-effectiveness compared to gold and silver nanoparticles, and the natural presence of various forms of copper in the human body [13,14].

Antimicrobial action is carried out by CuNPs by disrupting the cell membranes causing bacterial death. Dissolved copper ions bind to proteins and enzymes and interrupt their functions. Copper ions can cause oxidative stress in bacteria by the production of reactive oxygen species (ROS). Oxygen radicals damage proteins, lipids and nucleic acids, leading to the apoptosis of bacterial cells and destroying the bacteria [15]. CuNPs can also promote angiogenesis (formation of new blood vessels) by increasing the production of vascular endothelial growth factor (VEGF), improving blood supply and, thus, leading to repair and tissue regeneration. Moreover, copper and CuNPs enhance the expression of fibrinogen and collagen formation genes, which play a crucial role in wound healing [16].

Several studies demonstrated significant toxicity of CuNPs and Cu-ions, which limited their application as an antibacterial agent for wound treatment. Lan Song et al. demonstrated that the particle forms of CuNPs in suspensions highly contribute to toxicity in all exposed cell lines whereas copper ions (Cu^2+^) only cause significant responses in mammalian cell lines, indicating the species-specific toxicity of CuNPs. They demonstrated that the morphologies, the ion release rate of NPs, and the species-specific vulnerability of cells should all be considered during the application of CuNPs [17]. In addition, H. Karlsson [18] demonstrated that CuNPs can cause hemoglobin aggregation/precipitation and membrane damage because of the metal release process. Also, oxidative stress is described as the main mechanism of CuNPs toxicity [19]. Most research suggests that keeping a balance between the antibacterial effectiveness and the toxic effect of CuNPs by combining them with other active agents that could decrease toxicity and provide more effective antibacterial action.

Several studies have demonstrated that the combination of CuNPs with electrospun membranes can be used to develop effective solutions for wound treatment. Polyacrylonitrile (PAN)-based nanofibers with varying concentrations of CuNPs have shown antibacterial activity against bacteria resistant to highly effective antibiotics, indicating their potential for use on contact surfaces at risk of bacterial infections [20]. Additionally, CuNPs have been used to modify polyethylene oxide nanofibers with defensin peptides, showing high effectiveness with moderate toxicity in both in vitro and in vivo studies [21]. Another study highlighted the in vivo antibacterial and wound-healing effectiveness of PVA/PCL-based electrospun nanofibers with CuNPs and Quercus infectoria extracts [22] or silver nanoparticles [23]. In all cases, CuNPs were combined with additives to reduce their toxicity. However, these complex mixtures limit the widespread application of these solutions due to their complexity and high cost.

Our previous data demonstrated an effective approach to developing electrospun membranes using a mixture of polylactic acid (PLA) or polycaprolactone (PCL) with chitosan. These materials exhibited high biocompatibility and significantly enhanced wound healing [24,25]. Additionally, we demonstrated a notable hemostatic effect of chitosan-based electrospun membranes [26]. Given that chitosan can enhance antibacterial effects, we hypothesized that combining PLA and chitosan nanofibers with CuNPs impregnation could provide an effective solution for treating infected wounds while reducing the toxicity of CuNPs.

## 2. Materials and Methods

### 2.1. Materials

The low-molecular-weight chitosan powder (890,000 Da) was obtained from Glentham Life Sciences in Corsham, United Kingdom (CAS 9012-76-4). The 1.0 M acetic acid solution (CAS 7732-18-5) was purchased from Honeywell in Charlotte, NC, USA. The other reagents used in this study were sourced from Sigma-Aldrich in St. Louis, MO, USA, including Poly(L-lactide) powder (average Mn 40,000, CAS 26161-42-2), Poly(ethylene oxide) powder (average Mv ~300,000, CAS 25322-68-3), Polyethylene Glycol (MW 1500, CAS 25322-68-3), chloroform (≥99%, CAS 67-66-3), ethyl alcohol (≥99.8%, CAS 64-17-5), and NaOH (CAS 1310-73-2). All nutrient media, such as Muller–Hinton agar, Muller–Hinton broth, MacConkey agar, mannitol-salt agar, and cetrimide agar, were obtained from Sigma-Aldrich (Sigma-Aldrich, St. Louis, MO, USA).

### 2.2. Electrospun Patches Synthesis and Structural Assessment

First, 10 mL of 99.9% acetic acid was diluted with distilled water to a final concentration of 50% and adjusted to a total volume of 20 mL. This solution was mixed with 1.6 g of chitosan powder. Next, 1.6 g of polyethylene oxide (PEO) was added, and the mixture was thoroughly stirred. Separately, 0.2 g of polylactic acid (PLA) was dissolved in 5 mL of chloroform, and the excess chloroform was evaporated. This PLA solution was combined with the chitosan mixture, and then 1.2 g of polyethylene glycol (PEG) was added and prepared in the same way. The electrospinning parameters were detailed in our previous study [25]. The developed as-spun membranes were treated with a 1M sodium hydroxide (NaOH) solution (70% ethanol/30% water) for 12 h, thoroughly washed with distilled water, and left to dry overnight at room temperature.

The electrospun CH/PLA nanofiber membranes were functionalized with copper nanoparticles (CuNPs) supplied by Nano Pure Co., Wrocław, Poland. The CuNPs were incorporated into the CH/PLA membranes via drop-coating at a concentration of 100 µg/mL. After coating, the samples were air-dried for 24 h at room temperature [27].

Each electrospun membrane was observed using scanning electron microscopy (SEM) (Phenom ProX, Phenom-World BV, Eindhoven, The Netherlands), which was equipped with an energy-dispersive X-ray spectrometer (EDX). The average diameter of fibers was evaluated based on SEM micrographs using the Fiji software (ImageJ 1.51f; Java 1.8.0_102) [28]. Fibers were randomly chosen from three electrospun membranes of each type of sample (100 fibers from each specimen). Fiber diameters are reported as average values with their standard deviation.

### 2.3. Investigation of Antibacterial Properties

Bacterial strains of *S. aureus* B 918, *Escherichia coli* B 926 and *Pseudomonas aeruginosa* (*P. aeruginosa*) 27,853 were used in this experiment. For the cultivation, bacterial isolation and identification, Muller–Hinton agar, Muller–Hinton broth, MacConkey agar, mannitol-salt agar and cetrimide agar were used (Sigma-Aldrich, St. Louis, MO, USA). *Escherichia coli* and *Staphylococcus aureus*.

### 2.4. Bacteriological Experiment In Vitro

The selected bacterial strains (*S. aureus*, *E. coli* and *P. aeruginosa*) were grown in broth at 37 °C for 24 h. Membrane samples, each 0.5 cm^2^, were prepared under sterile conditions and placed into a sterile 24-well plastic plate containing 2 mL of a previously prepared bacterial suspension (10^5^ CFU/mL). As a control, untreated bacteria were suspended in nutrient broth. Following incubation periods of 2, 4, and 6 h, 10 µL samples from each well were transferred onto agar plates, which were then incubated at 37 °C overnight to count the surviving bacteria [29].

### 2.5. In-Vivo Experiment Design

For this study, 12-week-old Wistar male rats weighing 200–220 g were used. The laboratory animals were kept in the Vivarium (animal house) of Sumy State University (Sumy, Ukraine). Animal housing and all procedures were conducted in accordance with Directive 2010/63/EU of the European Parliament and the Council and were approved by the Commission on Bioethics Compliance in Experimental and Clinical Research of Sumy State University.

The experimental animals were housed separately in individual cages with a 12-h light/dark cycle at 25 °C and stable humidity. The rats had access to standard balanced pellets and tap water ad libitum. Animals were fasted for 12 h prior to the experimental procedures.

Under intravenous anesthesia with 10 mg per kg of medetomidine hydrochloride (“Prosedan”, Farmaton, Rivne, Ukraine), the interscapular areas of the experimental animals were shaved using a safe animal shaver and disinfected with 70% ethanol. A sterile wound defect (1.0 cm × 1.5 cm) was created with a sharp scalpel. The wound was infected with a gauze swab soaked in a bacterial mixture of *S. aureus*, *E. coli*, and *P. aeruginosa* (5 × 10^9^ CFU/mL). The swab was placed into the wound defect, sewn up, and removed after 72 h [30].

The animals were randomly divided into 4 groups based on the type of wound treatment used (Table 1).

A sterile dressing material was applied to the wound according to the treatment protocol and changed daily under aseptic conditions. The monitoring and assessment of the wound size were performed daily. The images of the wound area were measured using the Image-J version 1.52a software (Wayne Rasband, Kensington, MD, USA).

The rats were terminated by anesthesia overdose (medetomidine hydrochloride (“Prosedan” Farmaton, Ukraine) 70 mg per kg) on the 3rd, 14th, and 21st days of the treatment. Samples from the skin were collected for histology and immunohistochemistry.

### 2.6. Microbiological Assessment of the Wound

Quantitative and qualitative microbiological evaluations were performed using swabs of the wound surface and exudate. The first smear was collected 72 h after the application of the gauze swab and before the treatment. Subsequent smears were collected on the 3rd, 5th, and 7th days of the wound treatment. Smears were taken from both the central and peripheral parts of the wounds. Microbiological analysis of bacterial contamination was conducted using selective media, including MacConkey agar, mannitol-salt agar, and cetrimide agar, employing the streak plate technique. Bacterial plates were incubated at 37 ± 1 °C for 24 h, and the number of colony-forming units (CFU) was calculated [30].

### 2.7. Histological and Immunohistochemical Assessment

The skin tissue was fixed in 10% neutral buffered formaldehyde (Sigma-Aldrich, St. Louis, MO, USA), dehydrated using an ethanol gradient, and saturated with paraffin in a tissue processor (AT1010-EKA, Mariupol, Ukraine). Paraffin blocks were embedded using the ES5-EKA embedding station (Mariupol, Ukraine). Serial sections of 4 µm thickness were prepared with a Shandon Finesse 325 rotary microtome (Thermo Scientific, Waltham, MA, USA) and attached to adhesive SuperFrost microscopy slides (Thermo Scientific, Waltham, MA, USA).

The samples were stained with Mayer’s hematoxylin and eosin (BioGnost, Zagreb, Croatia) and mounted with a permanent mounting medium (Master Diagnostica, Granada, Spain).

Immunohistochemistry was performed according to the standard protocol using a Master polymer plus detection system (Peroxidase) (Master Diagnostica, Granada, Spain). Briefly, the tissue sections were deparaffinized, dehydrated, and subjected to antigen retrieval by heat incubation at 98 °C in 10 mM citrate buffer (pH 6.0) for 30 min. Endogenous peroxidase activity was then blocked, and the samples were incubated with a blocking agent. Following this, they were incubated with primary polyclonal antibodies: anti-CD68 (dilution 1:200, MyBioSource, San Diego, CA, USA), anti-CD163 (dilution 1:200, Master Diagnostica, Granada, Spain), and anti-MPO (dilution 1:200, Thermo Scientific, Waltham, MA, USA). The tissue samples were subsequently incubated with the HRP-polymer solution and visualized using an immunoperoxidase DAB kit (Master Diagnostica, Granada, Spain). The nuclei were counterstained with Mayer’s hematoxylin, and the samples were mounted with a mounting medium (Master Diagnostica, Granada, Spain) [31].

## 3. Results

### 3.1. Patches Structure

**Figure 1:**
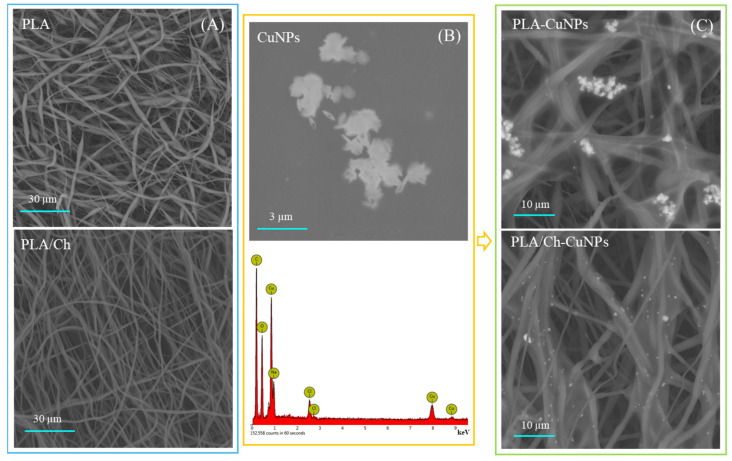
SEM and EDX analyses of the electrospun fibers. (**A**) PLA and PLA/Ch as spun fibers; (**B**) CuNPs morphology (upper row) with EDX analyses (down row); (**C**) PLA and PLA/Ch fibers enriched with CuNPs.

Both PLA and PLA/Ch patches are composed of randomly oriented fibers that form interconnected pores, as depicted in Figure 1. The fibers from both the PLA/Ch and PLA patches have similar average diameters, with the neat PLA fibers measuring 171 ± 120 nm and the the PLA/Ch blend fibers measuring 175 ± 63 nm. The addition of chitosan (Ch) to the electrospinning process positively affects the fiber size distribution, resulting in more uniformly sized fibers. The CuNPs are visualized as agglomerated particles with needle-like structures. The EDX analysis confirms the copper nature of these nanoparticles and their presence on the loaded membranes, demonstrating the successful incorporation of CuNPs into the electrospun nanofiber structures. The SEM images reveal that the CuNPs are well-distributed on the fiber surfaces of both PLA-CuNP and PLA/Ch-CuNP membranes, appearing as bright spots, which indicates the successful loading of the nanoparticles onto the electrospun membranes. This integration of CuNPs onto the nanofiber surfaces enhances the functional properties of the membranes, making them potentially useful for biomedical applications.

### 3.2. Antibacterial Activity of Novel Electrospun Patches

**Figure 2:**
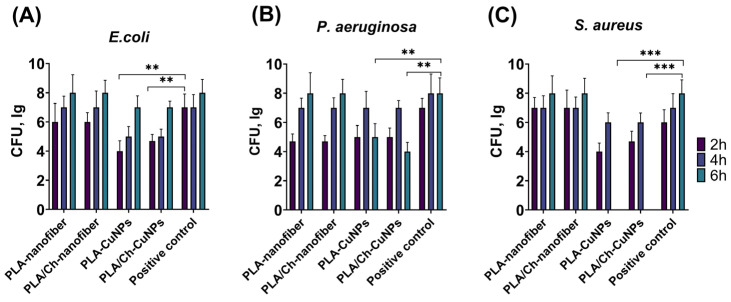
Dynamics of bacterial growth. Different electrospun membranes were incubated with different Gram-positive and Gram-negative bacteria for 2, 4, and 6 h: (**A**) *E. coli*, (**B**) *P. aeruginosa*, and (**C**) *S. aureus*. **—*p* < 0.01; ***—*p* < 0.001.

Our research data demonstrate that the addition of CuNPs significantly improves the antibacterial activity of both PLA and PLA/Ch nanofiber materials against *E. coli*, *P. aeruginosa* and *S. aureus* (Figure 2). PLA/Ch-CuNPs and PLA-CuNPs significantly inhibited *E. coli* growth after just 2, 4, and 6 h of exposure. Both PLA/Ch-CuNPs and PLA-CuNPs demonstrated antibacterial activity against *S. aureus* after 2 and 4 h. Notably, after 6 h, no *S. aureus* colonies were detectable on these membranes, while all other samples reached a bacterial density of Log 8 CFU. PLA/Ch-CuNPs and PLA-CuNPs membranes displayed significant inhibition of *P. aeruginosa* growth after 2 and 6 h compared to the other samples and the control.

These findings strongly suggest that PLA/Ch-CuNPs and PLA-CuNPs membranes possess a broad-spectrum antibacterial effect against both Gram-positive (*S. aureus*) and Gram-negative (*E. coli* and *P. aeruginosa*) bacteria. This promising result paves the way for their potential application as novel antibacterial agents, offering a significant advancement in the treatment of purulent skin wounds and reducing the burden of antibiotic-resistant infections. The incorporation of CuNPs not only enhances the antimicrobial properties of the nanofiber membranes but also maintains their structural integrity and biocompatibility, making them suitable for use in various biomedical applications.

### 3.3. Wound Size Evaluation

**Figure 3:**
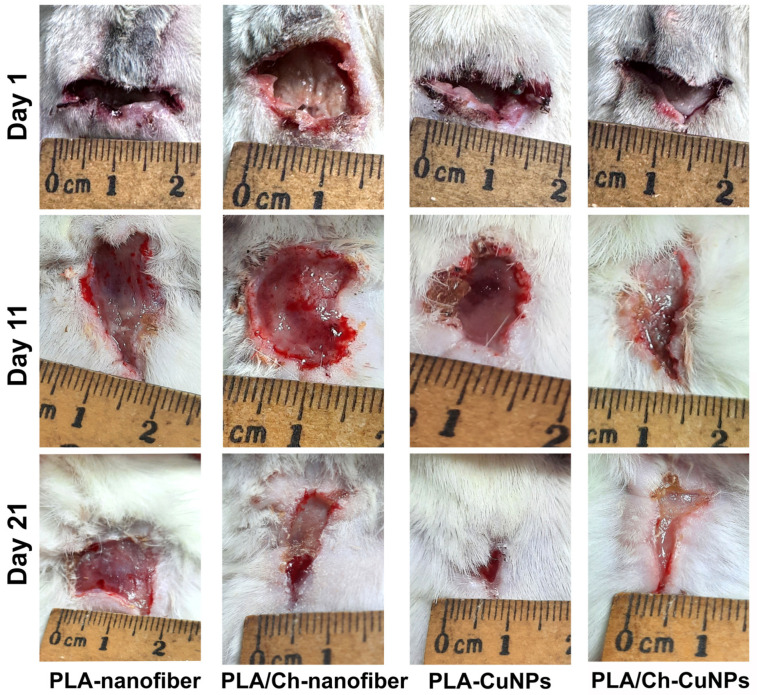
Wound healing process in the laboratory animal model. The wound defect was treated with different patches applied between day 1 and day 21 (images of additional days are represented in Figure A1).

All animals developed purulent inflammation on the wound surface following the application of the gauze swab inoculated with the bacterial strain mixture. At 72 h post-application, the intensity of the primary inflammatory response was consistent across all groups. Wound healing was assessed by measuring the wound area every second day (Figure 3). From day 1 to day 7 of the wound treatment, all experimental groups exhibited an increase in the wound area, indicative of an acute inflammation progression and the predominance of destructive processes within the wound.

**Figure A1:**
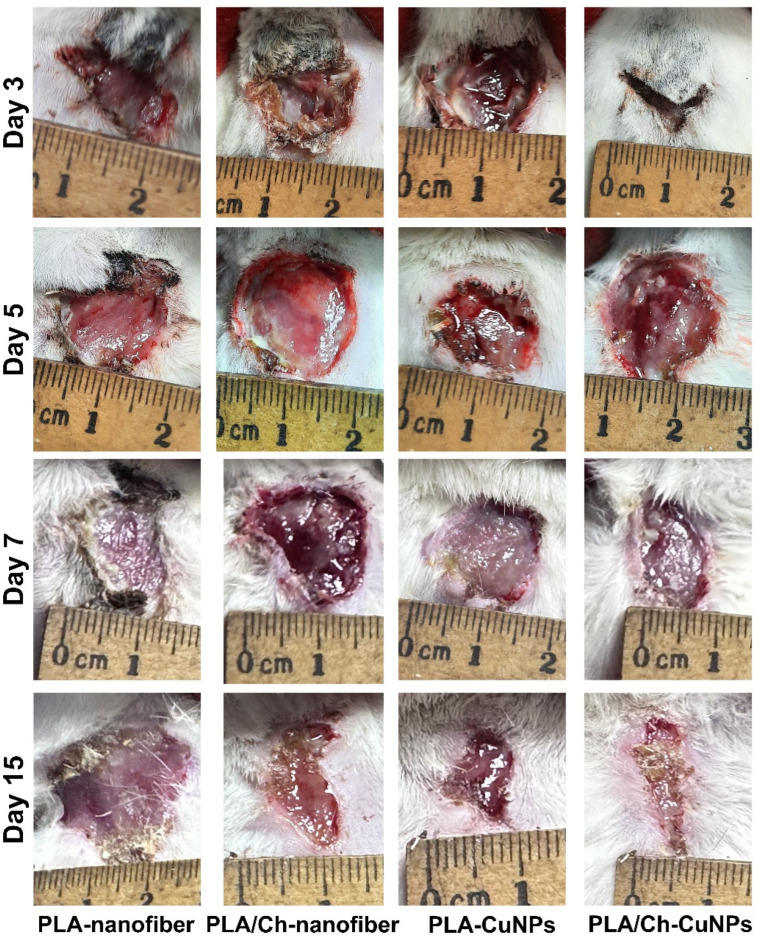
Wound defects in the laboratory animals with different treatments throughout the experiment.

By day 9, wound size dynamics shifted, with the wound area gradually decreasing at different rates among the experimental groups (Figure A1). Notably, from day 9 onwards, the group treated with PLA/Ch-CuNPs showed the smallest wound area. Conversely, the PLA-nanofiber-treated group exhibited no signs of wound healing by day 21, with the wound area remaining unchanged from the start of the experiment.

**Figure 4:**
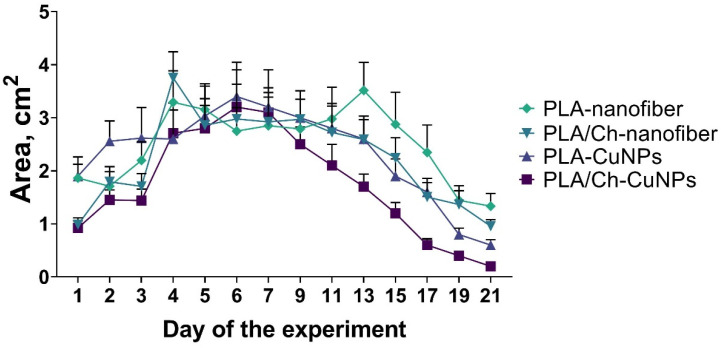
Dynamic of wound size in the laboratory animals. The size of the wound defect was measured daily within 21 days of the treatment.

No significant differences were observed between the PLA-CuNPs and the PLA/Ch treated groups, both of which exhibited similar wound healing dynamics and trends (Figure 4). However, the healing rate in these groups was lower than that observed in the PLA/Ch-CuNPs treated group but higher than that in the PLA-nanofiber-treated group. These findings suggest that the incorporation of CuNPs into PLA/Ch nanofiber materials significantly enhances wound healing, potentially due to their broad-spectrum antibacterial properties and the creation of a conducive environment for tissue regeneration.

### 3.4. Microbiological Evaluation

**Figure 5:**
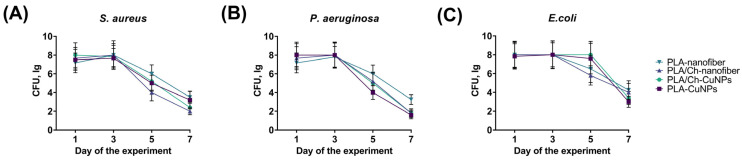
Microbiological composition of the wound. The graphs show the bacterial contamination of the wound in experimental animals at different time points of the treatment by the following bacteria: (**A**) *S. aureus*. (**B**) *P. aeruginosa.* (**C**) *E. coli*.

Evaluation of the wound microbiota was performed on the 1st, 3rd, 5th, and 7th days of treatment, revealing that different treatments affected bacterial populations within 3 to 7 days (Figure 5). The CFU count of *S. aureus* remained stable in all animals up to the 3rd day of the experiment, but gradually decreased in all groups on the 5th and 7th days of treatment (Figure 5A). The highest CFU counts for *S. aureus* on day 7 were observed in the PLA-nanofiber and PLA-CuNPs groups, while the lowest counts were noted in the PLA/Ch and PLA/Ch-CuNPs treated groups.

A similar trend was observed for *P. aeruginosa* (Figure 5B). However, the PLA-nanofiber dressing material exhibited the lowest efficacy, whereas all other dressings demonstrated similar antimicrobial activity. The CFU counts of *E. coli* remained stable up to the 5th day of treatment, with a tendency to decrease only on day 7 across all animal groups (Figure 5C). The PLA/Ch and the PLA-nanofiber groups showed the lowest antimicrobial activity, while the other wound dressings demonstrated comparable efficacy in inhibiting *E. coli* growth in the wounds.

### 3.5. Histological and Immunohistochemical Assessment

Histological assessment of the dressing materials’ effectiveness with different compositions was performed on the 3rd, 14th, and 21st days of the experiment. The development of experimental purulent wounds followed general regularities and stages in all four groups of rats. However, there were differences in the histological and immunohistochemical indicators of tissue damage, the degree of repair, and the level of inflammatory and exudative changes.

**Figure 6:**
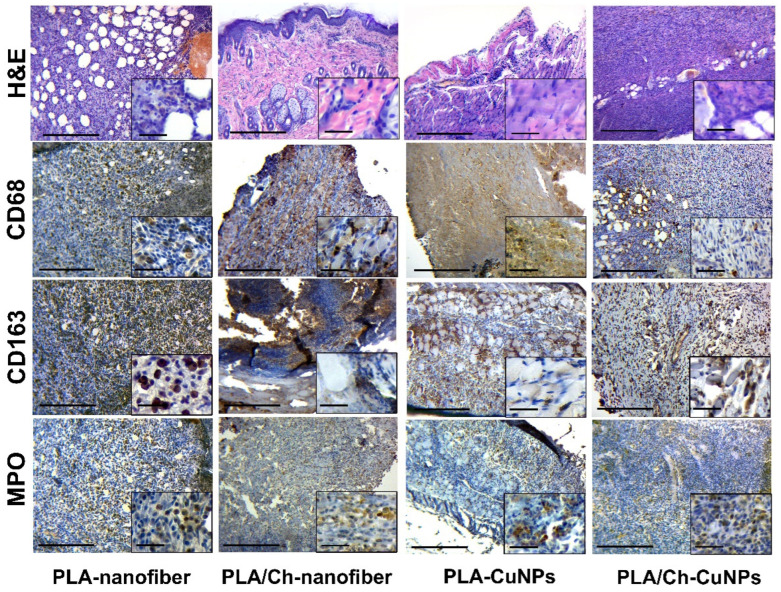
Histological and immunohistochemical study of the skin tissue samples from experimental animals on the 3rd day of the treatment. Magnification of the main image corresponds to ×100 (scale bar is equal to 200 µm), and magnification of the insert corresponds to ×400 (scale bar is equal to 25 µm).

On the 3rd day of wound development in the PLA-nanofiber-treated group, we observed signs of purulent inflammation with interstitial edema and the formation of immature granulation tissue (Figure 6). Numerous newly formed blood vessels were present in the skin tissues, which were expanded with hemorrhages. No remnants of the dressing material were detected in the wound defect.

During the same period, necrotic-inflammatory changes were more pronounced in the group treated with PLA/Ch-nanofiber. Additionally, remnants of chitosan from the bandage material, surrounded by a shaft of inflammatory infiltration, were observed in the wound.

The animals in the PLA-CuNPs treated group exhibited signs of acute purulent inflammation of the tissue walls and the bottom of the wound, with distinct exudative changes, such as interstitial swelling of all skin layers and blood vessel congestion, on the 3rd day of the experiment.

In samples from the PLA/Ch-CuNPs treated group, signs of acute purulent inflammation with a moderate level of exudation and the presence of immature granulation tissue with active inflammatory processes were observed on the 3rd day.

After 14 days of the experiment, we noted that acute inflammatory manifestations gradually subsided, and reparative changes accelerated in most cases. There was a transition from acute to chronic inflammatory changes, marked by a decrease in neutrophils and an increase in histiocytes, macrophages, and fibroblasts.

**Figure 7:**
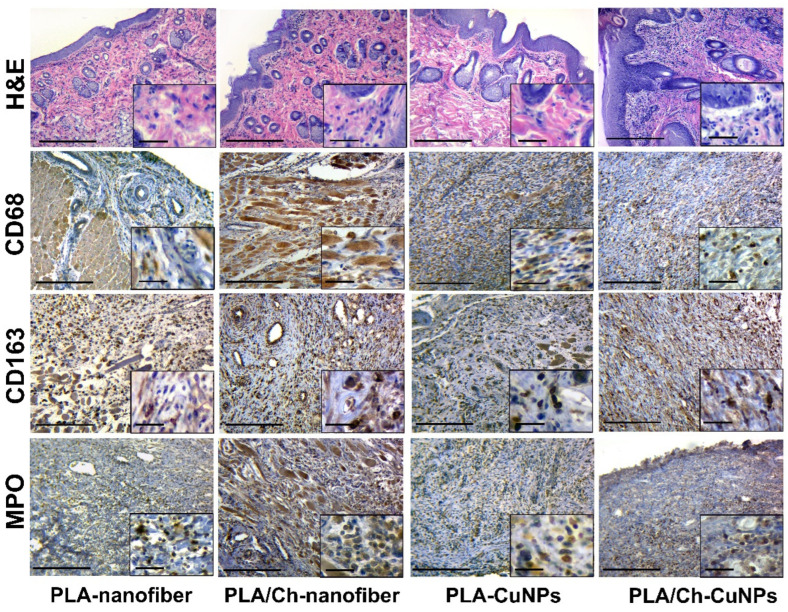
Histological and immunohistochemical study of the skin tissue samples from experimental animals on the 14th day of the treatment. Magnification of the main image corresponds to ×100 (scale bar is equal to 200 µm), and magnification of the insert corresponds to ×400 (scale bar is equal to 25 µm).

Skin samples from the PLA-nanofiber treated group contained granulation tissue of varying maturity, with persistent inflammatory infiltration and swelling of skin tissues (Figure 7). Tissues adjacent to the wound showed signs of edema and dyscirculatory disorders, and fibrous tissue was found in the dermis.

Samples from the PLA/Ch-nanofiber treated group exhibited granulation tissue of varying maturity, with moderate inflammation and edema after 14 days of treatment.

During the same period, samples from the PLA-CuNPs treated group demonstrated similar pathohistological effects in the wound, including the presence of granulation tissue of varying maturity, signs of inflammation, and edema.

In the PLA/Ch-CuNPs treated group, there was a decrease in inflammatory and edematous tissue symptoms, with an increase in reparative-replacement processes, such as the development of granulation tissue and collagen fibers, after 2 weeks of treatment.

**Figure 8:**
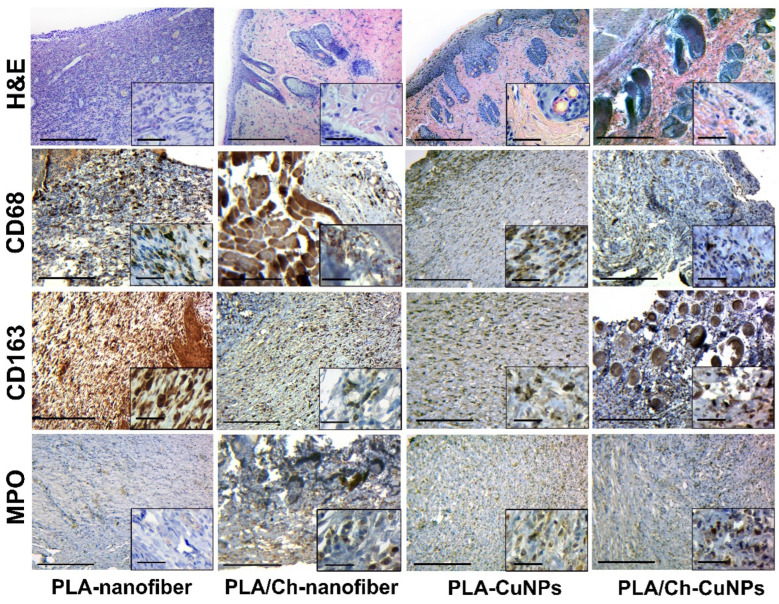
Histological and immunohistochemical study of the skin tissue samples from experimental animals on the 21st day of the treatment. Magnification of the main image corresponds to ×100 (scale bar is equal to 200 µm), and magnification of the insert corresponds to ×400 (scale bar is equal to 25 µm).

During the 21-day experiment, a tendency for the inflammatory process to attenuate, the granulation tissue to mature, and the scar tissue to form (fibrillization of the wound) was observed in all studied groups. However, each type of dressing material had distinct characteristics. Wound treatment with PLA-nanofiber dressing material showed persistent inflammation (mostly insignificant and moderate), a tendency toward chronicity, and the presence of immature granulation tissue remnants (Figure 8).

In the PLA/Ch-nanofiber treated group, a significant amount of connective tissue and remnants of mature granulation tissue with weak inflammatory infiltration was observed after 21 days of treatment. Treatment with PLA-CuNPs revealed the presence of immature granulation tissue remnants, small focal inflammatory infiltrates, and moderate swelling in the wound. After 21 days of treatment by PLA/Ch-CuNPs, the following changes were observed in the wound: connective tissue with remnants of mature granulation tissue, moderate edema, and small inflammatory infiltration.

An important aspect of studying the possible resorptive toxic effect of copper compounds from the dressing material is the study of structural changes in the tissues of the internal organs of experimental animals using histology. To control the possible resorptive effect of copper ions from nanoparticles, we took tissues of internal parenchymal organs that are very sensitive to the effects of toxins, such as the heart, kidneys, and liver [32].

**Figure A2:**
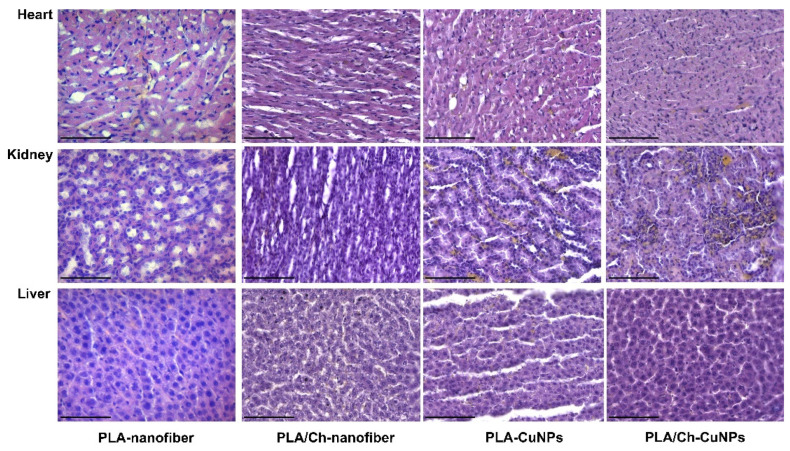
Histological evaluation of internal organs after application of different dressing materials for infected wound healing on the 3rd day after treatment initiation. Magnification of the image corresponds to ×400 (scale bar is equal to 100 µm).

**Figure A3:**
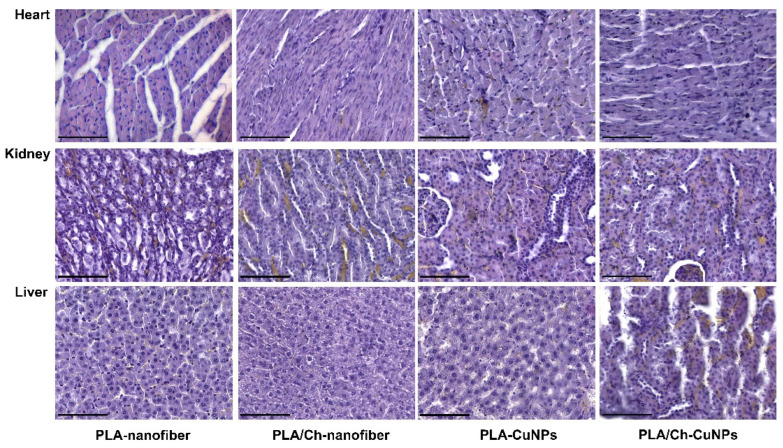
Histological evaluation of internal organs after application of different dressing materials for infected wound healing on the 14th day after treatment initiation. Magnification of the image corresponds to ×400 (scale bar is equal to 100 µm).

**Figure A4:**
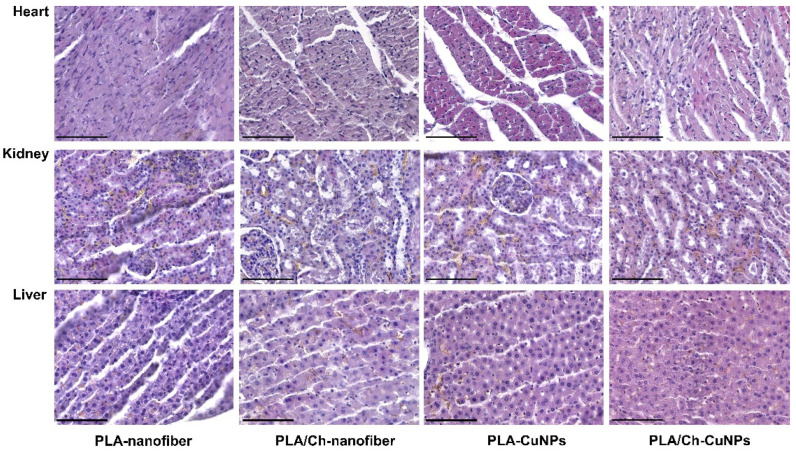
Histological evaluation of internal organs after application of different dressing materials for infected wound healing on the 21st day after treatment initiation. Magnification of the image corresponds to ×400 (scale bar is equal to 100 µm).

During a detailed study of the histological structure of the internal parenchymal organs, sensitive to toxic changes, we did not find a distinct resorptive adverse effect on their structure. Pathohistological changes were stereotyped and did not directly depend on the state and activity of the modeled wound process and the composition of medical dressings. Thus, typical changes consisted of the presence of edema of the connective tissue; the expansion of the distance between the parenchymal cells of the myocardium, kidneys, or liver; and the presence of focal dystrophic changes in individual cells or groups of cells with the accumulation of small fat droplets or protein granules. Common phenomena were the presence of a plethora of small and medium vessels and the formation of isolated hemorrhages (Figure A2, Figure A3 and Figure A4). These changes, most likely, had individual specificity, depending on the state of the animal’s body and the specifics of the reaction of tissues to the procedures of taking and processing material for histological examination. We conclude this because the patterns of histological changes did not depend on the period of observation and the composition of the dressing material.

The skin tissues of rats with experimentally induced purulent wounds are characterized by inflammatory cell infiltration, a crucial indicator of the pathological process. This typical pathological process can be qualitatively and quantitatively assessed through immunohistochemical studies of pro-inflammatory (M1) and anti-inflammatory (M2) macrophage markers [33]. The activity and the acute phase of inflammation are characterized by the number of neutrophils in the inflammatory infiltrate [34].

The immunohistochemical marker CD68, which has cytoplasmic staining in cells, is characteristic of pro-inflammatory M1 phenotype macrophages that perform protective and scavenger functions [35]. In the wound tissue, a large number of M1-type macrophages are found among the debris, necrotic tissue, immature granulation tissue, and inflammatory infiltrates.

The marker CD163 also has cytoplasmic staining and is used to identify anti-inflammatory M2 phenotype macrophages. This phenotype has immunosuppressive and proliferative effects and is a constant element of the tumor microenvironment. It is expected that the number of M2 phenotype macrophages will increase with the enhancement of reparative processes and the decrease of inflammation intensity [36].

The immunohistochemical study of CD68- and CD163-positive cell expression has limitations and caveats. Its main issue is the positive overlapping of these markers with some cell populations, such as fibroblasts, endothelial cells, and pericytes [37].

The MPO marker, or myeloperoxidase, is the main enzyme of neutrophils and, thus, a convenient means of their immunohistochemical identification [38]. A large number of neutrophils is a direct sign of acute purulent inflammation, while even a small number of neutrophils in the inflammatory infiltrate indicates the activity of the inflammatory process [34].

**Figure 9:**
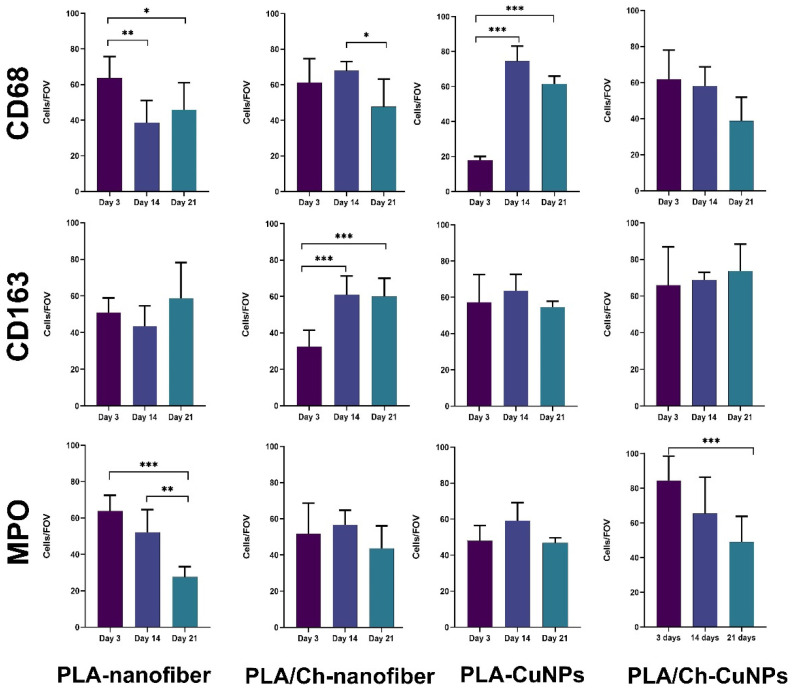
The evaluation of the cellular composition in the inflammatory infiltrate in the tissue samples from experimental animals. Skin tissue samples were examined immunohistochemically with further quantitative evaluation of CD68+, CD163+, and MPO+ immune cells at the different time points of the treatment. *—*p* < 0.05; **—*p* < 0.01; ***—*p* < 0.001.

Monitoring the dynamics of CD68-positive cells in wound tissues revealed that in the groups treated with PLA nanofiber and combined PLA nanofiber, chitosan, and copper nanoparticles, the number of pro-inflammatory M1 macrophages decreased from the beginning to the end of the experiment (Figure 9). In the PLA/Ch-treated group, an increase in macrophage numbers was observed at 14 days, possibly due to macrophage activation for chitosan degradation. In the copper nanoparticle-treated group, low initial macrophage activity was followed by a significant increase, possibly due to the influence of copper ions on macrophage activity [39,40].

The dynamics of CD163-positive cells and the M1/M2 macrophage phenotype ratio followed a stereotypical pattern in almost all groups: M1 macrophages predominated at the beginning of the experiment during acute inflammation and resorptive activity, while the activation of proliferative and reparative processes after 14 days showed a predominance of M2 phenotype macrophages. Deviations from this pattern were observed only in the group treated with PLA nanofibers and copper nanoparticles, where overall macrophage activity was initially suppressed, with a partial recovery of typical reactivity in later stages, suggesting a delayed standard response to injury. This may be attributed to the toxic effects of copper ions, indirectly confirmed by the intense necrotic exudative reaction of the wound tissues in the early observation period and subsequent organism adaptation [41].

Neutrophil activity in the modeled purulent skin wound of rats, treated with various compositions of dressing materials, generally followed the logical progression of the inflammatory process in the wound. Acute tissue inflammation arose at the experiment’s onset, decreasing during wound cleaning and healing. Again, an exception to the general pattern was the group with PLA nanofiber and copper nanoparticles. In this group, a delayed increase in neutrophil numbers was observed on day 14, with suppressed reactivity at the first control point of 3 days. Subsequently, reactivity to purulent inflammation adapted, and the normal course of events resumed with slightly delayed wound healing.

As observed, the application of dressing materials composed of various components such as PLA nanofibers, copper nanoparticles, and chitosan did not result in a radical improvement in the treatment of purulent wounds. However, detailed examination of the experimental results highlights the specific impact of each component on the wound-healing process. It is evident that each component has its advantages and disadvantages, making the search for their optimal combination a justified endeavor that could yield successful outcomes.

The use of dressing material composed solely of PLA nanofibers (serving as the control group) demonstrated an active exudative process of moderate intensity and reparative changes with fibrous and inflammatory components in the wound. This indicates a lack or weakness of antibacterial activity of the material, resulting in inflammatory and necrotic damage, prolonged healing, and fibrosis. This component of wound dressing could be used only as a mechanical supporting part or a drug-loading component.

The dressing material combining PLA nanofibers with chitosan showed interesting results. Despite pronounced inflammatory and necrotic changes in the wound at the beginning of the application, significant improvement in the wound healing process was observed subsequently. It appears that the addition of chitosan reduces exudative and edematous changes in the tissues, likely due to its absorbent/hygroscopic and associated antibacterial properties [42]. However, it is essential to consider the potential for chitosan to provoke a “foreign body” reaction, leading to macrophage activity and the formation of neutrophilic walls around it. Ultimately, after wound cleaning, the healing process continued quite effectively, albeit with increased tissue fibrosis as a consequence.

The observations of the PLA nanofiber dressing material with added copper nanoparticles indicate that copper-containing compounds have a suppressive effect on the inflammatory cellular response (cellular immunity) at the initial stage of application. Interestingly, there was a reduction in both pro-inflammatory and anti-inflammatory macrophages, with an overall low level of neutrophils against a backdrop of necrotic tissue changes. This suggests the presence of toxicity at the applied concentration of copper ions released from the nanoparticles, indicating the necessity to reduce the concentration of nanoparticles in such dressings.

The most promising results in terms of pathohistological changes were observed with the wound dressing combining PLA nanofibers as the woven base with chitosan and copper nanoparticles. This combination of the mechanical protection of PLA nanofibers, the absorbent properties of chitosan, and the antibacterial activity of copper nanoparticles reduced exudative manifestations in the wound and promoted faster healing compared to the other groups. The PLA base and chitosan are biodegradable, with chitosan potentially mitigating the toxic effects of copper ions, possibly through adsorption [43].

The application of copper nanoparticles for external wound treatment presents a major challenge due to the high toxicity of copper ions. However, the potential solution lies in combining them with other compounds and optimizing the dose of the active substance. Determining the correct proportions and achieving a controlled, stable release of optimal copper ion concentrations could result in an inexpensive dressing material with high antibacterial activity for the treatment of purulent wounds.

## 4. Conclusions

This study explored the development and application of novel electrospun wound dressings based on PLA and Chitosan with CuNPs incorporation. From the different combinations, the PLA/Ch-CuNPs dressing material exhibited the most promising results, showing significant antibacterial activity against both Gram-positive (*S. aureus*) and Gram-negative (*E. coli* and *P. aeruginosa*) bacteria, as well as superior wound healing properties compared to the other tested materials. The combination of mechanical protection provided by PLA nanofibers, the adsorptive and antimicrobial properties of chitosan, and the antibacterial efficacy of CuNPs contributed to reduced exudative manifestations and accelerated healing.

However, this study found the potential toxicity of copper ions at certain concentrations, indicating the need for careful optimization of CuNP dosage to balance antimicrobial activity and biocompatibility. These findings suggest that the inclusion of chitosan may help mitigate the toxic effects of copper ions, potentially through adsorption.

Overall, the integration of CuNPs into electrospun nanofiber membranes offers a promising approach for enhancing the antibacterial and wound-healing capabilities of dressing materials. Future research should focus on optimizing the composition and concentration of these components to maximize their therapeutic potential while minimizing the adverse effects. With further refinement, these advanced wound dressings could provide an effective and cost-efficient solution for the treatment of purulent skin wounds, addressing the growing challenge of antibiotic resistance and chronic inflammation.

## Figures and Tables

**Table 1:** Four groups of animals treated with different dressing material at different concentrations of CuNPs.

Group Number	Dressing Material	Concentration of CuNPs, µg/mL
1	PLA-nanofiber	0
2	PLA/Ch-nanofiber	0
3	PLA-CuNPs	100
4	PLA/Ch-CuNPs	100

## Data Availability

Data are available upon request from the corresponding authors.
